# Confounding-aware disproportionality analysis reveals disease-inherent versus drug-attributable endocrine safety signals of immune checkpoint inhibitors

**DOI:** 10.3389/fphar.2026.1868030

**Published:** 2026-06-23

**Authors:** Osman Bilge Kaya, Refika Yorulmaz Kaya

**Affiliations:** 1 Department of Medical Oncology, Dr. Abdurrahman Yurtaslan Ankara Oncology Research and Training Hospital, Ankara, Türkiye; 2 Department of Endocrinology and Metabolism, Gazi University Faculty of Medicine, Ankara, Türkiye

**Keywords:** adrenocortical carcinoma, confounding bias, disproportionality analysis, FAERS, immune checkpoint inhibitors, immune-related adverse events (irAE), pharmacovigilance, thyroid carcinoma

## Abstract

**Background:**

Conventional disproportionality analysis assumes comparable background event rates between case and control cohorts. In patients with primary endocrine malignancies treated with immune checkpoint inhibitors (ICIs), this assumption fails: disease-inherent hormonal dysregulation and therapy-related sequelae inflate background event rates and may produce signals misattributed to ICI exposure. No prior study has used tumor-type-specific reference populations to separate drug-attributable from disease-inherent signals in this setting.

**Methods:**

Forty quarterly FAERS cycles (Q1 2015 to Q4 2024) were analyzed after deduplication of 2,264,070 reports. In a pooled analysis, 329 ICI-treated endocrine cancer cases were compared with 80,191 ICI-treated non-endocrine cancer controls across six pre-specified endocrine immune-related adverse event categories, with false discovery rate (FDR) correction. The central step was a confounding-aware reanalysis comparing ICI-exposed patients of a given tumor type against a reference cohort with the same malignancy but no ICI exposure, using logistic regression adjusted for age and sex. The thyroid carcinoma association was tested using reporter type, Firth penalized regression, multiple imputation, and a tipping-point analysis.

**Results:**

Pooled adrenal insufficiency did not reach the signal threshold after FDR correction (ROR = 1.922; 95% CI 1.051–3.513; q = 0.143). This non-significance concealed two opposing patterns. In ACC, adrenal insufficiency was the strongest uncorrected subgroup signal yet was not associated with ICI exposure after adjustment (OR = 0.58; 95% CI 0.09–1.95); with four exposed events and 12.6% power, this subgroup cannot confirm or exclude an association. In thyroid carcinoma, ICI exposure showed markedly higher adrenal insufficiency reporting odds (OR = 13.33; 95% CI 4.81–31.85), remaining positive under reporter adjustment, Firth regression, and multiple imputation, with a tipping-point analysis indicating only implausibly extreme confounding could nullify it.

**Conclusion:**

Pooled pharmacovigilance can misclassify drug-attributable and disease-inherent events. Tumor-type-specific reference modeling separates them: ACC data are most consistent with disease-inherent pathophysiology, whereas thyroid carcinoma shows a robust adrenal insufficiency signal. We interpret the thyroid signal as hypothesis-generating, since differential endocrine surveillance may contribute to its magnitude, and prospective validation is warranted.

## 1 Introduction

Immune checkpoint inhibitors (ICIs) have reshaped oncologic practice by targeting co-inhibitory receptors including cytotoxic T-lymphocyte-associated protein 4 (CTLA-4), programmed cell death protein 1 (PD-1), and its ligand (PD-L1) (Sharma and Allison, 2015; Ribas and Wolchok, 2018). Alongside durable antitumor responses, checkpoint blockade produces a distinctive immune-related adverse event (irAE) profile that can affect virtually any organ system (Postow et al., 2018; Cukier et al., 2017). The endocrine system is among the most frequently and persistently involved, with ICI-associated endocrinopathies comprising thyroid dysfunction, hypophysitis, primary adrenal insufficiency, and immune-mediated diabetes mellitus, often requiring lifelong hormone replacement (Chang et al., 2019; Wright et al., 2021; Yoo et al., 2023). Combination anti-CTLA-4 plus anti-PD-1 therapy carries the highest endocrine irAE risk relative to monotherapy (Barroso-Sousa et al., 2018), and severe manifestations such as adrenal crisis and diabetic ketoacidosis are readily misattributed to the underlying cancer or its treatment (Stelmachowska-Banaś and Czajka-Oraniec, 2020).

Spontaneous reporting databases such as the FDA Adverse Event Reporting System (FAERS) are the principal infrastructure for post-marketing ICI safety surveillance. Signal detection in these databases relies on disproportionality analysis, which compares the reporting rate of an event in the drug of interest against a reference cohort. The validity of this approach rests on a critical assumption: that the background prevalence of the adverse event is comparable between case and control cohorts. When background prevalence diverges systematically between populations, disproportionality metrics conflate drug-attributable signal with disease-inherent confounding and may yield quantitatively impressive but causally misleading associations.

Patients whose primary diagnosis is an endocrine malignancy violate this assumption in structural ways. In adrenocortical carcinoma (ACC), autonomous glucocorticoid hypersecretion and adjuvant mitotane-induced adrenolysis establish a high background prevalence of adrenal insufficiency independent of any ICI exposure (Wright et al., 2021; Terzolo et al., 2007). Thyroid carcinoma patients frequently enter pharmacovigilance databases already carrying post-thyroidectomy or post-radioiodine hypothyroidism. These disease- and treatment-inherent features distort background rates of endocrine outcomes in predictable, tumor-type-specific ways, and conventional pooled disproportionality analysis is structurally unable to separate such confounding from genuine drug-attributable signals. The clinical cost of this methodological limitation is twofold: genuine ICI toxicity may be obscured by disease noise, while disease-inherent endocrine dysfunction may be spuriously attributed to ICI exposure, with downstream consequences for clinical decision-making, treatment discontinuation, and regulatory signal adjudication.

Several strategies have been proposed to mitigate confounding in spontaneous reporting analyses, including restriction to within-class active comparators, stratification by indication, and the use of disease-matched reference groups, all of which aim to replace the self-referential whole-database denominator with a more clinically comparable one. These approaches are well developed in formal pharmacoepidemiology but have been applied only sparingly to endocrine irAE signal detection in cancer populations. To our knowledge, no prior pharmacovigilance study has addressed this gap in ICI-treated endocrine cancer patients by applying tumor-type-specific ICI-naive reference populations with explicit demographic adjustment. We hypothesized that conventional signal detection in this population misclassifies disease-inherent endocrine dysfunction as ICI-attributable toxicity, and that a confounding-aware framework employing tumor-type-specific reference modeling would reveal clinically distinct signals that pooled analysis obscures. The present study accordingly aimed (i) to characterize ICI-associated endocrine irAE signals in patients with primary endocrine malignancies using 40 quarterly FAERS cycles (Q1 2015 to Q4 2024), (ii) to apply tumor-type-specific ICI-naive reference cohorts with multivariable demographic adjustment in order to distinguish drug-attributable from disease-inherent signals, and (iii) to model time-to-onset hazard trajectories using parametric survival analysis to inform monitoring strategy.

## 2 Materials and methods

### 2.1 Data source

This pharmacovigilance study used the FDA Adverse Event Reporting System (FAERS), a spontaneous post-marketing surveillance database. FAERS is administered by the U.S. Food and Drug Administration and receives reports primarily from U.S. healthcare professionals and consumers, with a smaller contribution of foreign reports submitted by pharmaceutical manufacturers. Adverse event terms were coded using MedDRA terminology (Sakaeda et al., 2013). As FAERS data are publicly available and fully de-identified, no ethics approval or patient consent was required.

### 2.2 Study period and data processing

Forty consecutive quarterly FAERS data files (Q1 2015 to Q4 2024) were downloaded from the FDA FAERS Quarterly Data Extracts portal (https://fis.fda.gov/extensions/FPD-QDE-FAERS/FPD-QDE-FAERS.html) on 3 April 2026. Records were deduplicated in two stages following FDA guidance: first, for each FAERS case number (CASEID) only the report with the highest follow-up version (latest FDA_DT, then highest PRIMARYID) was retained, removing superseded follow-up versions; second, residual duplicates were removed by matching on the combination of event date, age, sex, reporter country, and suspect-drug set. This yielded 2,264,070 unique reports prior to cohort selection. Age was missing in 43 case cohort patients (13.1%) and 17,114 controls (21.3%); sex was missing in 28 case patients (8.5%) and 9,650 controls (12.0%). Complete-case analysis was applied under a Missing at Random (MAR) assumption, namely, that missingness is conditionally independent of the outcome given observed covariates. This assumption is plausible in FAERS because documented missingness patterns largely reflect reporter-specific completeness practices, for example, consumer reports omitting demographic fields more often than physician reports, rather than case-level characteristics of the endocrine adverse event. Because MAR is formally untestable in spontaneous reporting data, its implications were examined directly through multiple imputation and a tipping-point analysis (Sections 2.5, 4). All analyses were performed in R 4.5.3 (2026-03–11 UCRT) using tidyverse (v2.0.0), survival (v3.7-0), epitools (v0.5-10.1), PhViD (v1.0.8), tableone (v0.13.2), logistf (v1.26.1), mice (v3.19.0), and pwr (v1.3-0).

### 2.3 Cohort definition

Two distinct comparator structures were used and should be distinguished. For the pooled disproportionality analysis, the case cohort comprised FAERS reports documenting a primary endocrine malignancy (adrenocortical carcinoma, thyroid carcinoma, pancreatic neuroendocrine tumor, pheochromocytoma/paraganglioma, or parathyroid carcinoma) with at least one ICI listed as a suspect drug, and the control cohort comprised ICI-exposed reports with non-endocrine oncologic indications. Endocrine malignancies and ICI exposure were identified from the indication and suspect-drug fields, respectively; a report contributed to a single tumor-type subgroup based on its recorded indication. ICI agents included nivolumab, pembrolizumab, cemiplimab, atezolizumab, durvalumab, avelumab, ipilimumab, and tremelimumab. Separately, for the confounding-aware analysis, tumor-type-specific ICI-naive reference cohorts were assembled from FAERS reports carrying the same endocrine malignancy but with no ICI listed among the suspect or concomitant drugs; these served as disease-matched background populations against which the ICI-exposed patients of the same tumor type were compared. The full cohort construction and analytical workflow, including the relationship between the pooled comparison and the tumor-type-specific reference design, is shown in Supplementary Figure S1.

### 2.4 Disproportionality analysis

Six pre-specified endocrine irAE categories were evaluated: adrenal insufficiency, thyroid dysfunction, hypophysitis, immune-mediated diabetes mellitus, hypoparathyroidism, and gonadal dysfunction. Each category was operationalized through a fixed set of MedDRA Preferred Terms applied uniformly across the pooled analysis and every tumor-type and drug-class stratum. The adrenal insufficiency term set comprised adrenal insufficiency, adrenocortical insufficiency including its acute form, adrenal failure, hypocortisolism, and primary adrenal insufficiency. The immune-mediated diabetes category captured type 1 and insulin-dependent diabetes mellitus together with diabetic ketoacidosis, so that autoimmune-type presentations were not omitted. Signal detection employed the reporting odds ratio (ROR), considered significant when the lower bound of the 95% CI exceeded 1.0 (van Puijenbroek et al., 2002), and the Bayesian information component (IC), considered significant when IC_025_ exceeded 0 (Bate et al., 1998). Fisher exact tests provided the p-values entering multiplicity correction, and Benjamini–Hochberg FDR correction was applied across a single pre-specified family of 45 comparisons spanning the quantitatively analyzable irAE categories within the pooled, tumor-subgroup, and drug-class strata (Benjamini and Hochberg, 1995). This 45-comparison family defined the confirmatory analysis. The tumor-type-specific confounding-adjusted regressions and their sensitivity analyses, together with the temporal trend analyses, were pre-specified as a separate set of focused, hypothesis-driven tests and were therefore not included in the 45-comparison FDR family; they are reported with their own confidence intervals and interpreted as confirmatory (for the confounding analyses) or exploratory and descriptive (for the temporal analyses), as indicated in the corresponding sections. Associations with FDR q < 0.05 within the comparison family were classified as significant; values in the q = 0.05 to 0.10 range were not treated as significant but were noted where relevant as not excluding a signal. Gonadal dysfunction, and hypoparathyroidism within most strata, yielded case counts too small for stable estimation and were reported as not estimable.

While addressing reviewer comments we identified that the acute adrenocortical insufficiency term had not been applied uniformly across all strata in an earlier working version of the analysis, which produced minor numerical discrepancies between interim tables. To remove this inconsistency we re-ran the entire disproportionality and confounding pipeline using the single adrenal term set defined above, applied identically to the pooled, drug-class, and tumor-type analyses. This standardization did not change the direction of any finding or any principal conclusion. The pooled adrenal insufficiency signal remained below the FDR significance threshold under both the earlier and the standardized definitions, ICI exposure remained non-significant in the demographically adjusted ACC subgroup, and the thyroid carcinoma association remained strong and robust across the sensitivity analyses. All tables and the archived analysis code were updated to reflect the standardized definitions.

### 2.5 Confounding analysis

For the purposes of this study, a subgroup signal was operationally classified as most consistent with a disease-inherent explanation when an uncorrected association attenuated toward or below the null after adjustment against the tumor-type-specific ICI-naive reference cohort, with the ICI-exposed and ICI-naive event rates of the same malignancy being similar; conversely, a signal was operationally classified as more consistent with a drug-attributable (and therefore hypothesis-generating) explanation when the ICI-exposed rate substantially exceeded the disease-matched ICI-naive rate and the adjusted association remained positive and robust to sensitivity analyses. These labels denote the explanation best supported by the disproportionality data rather than a confirmed causal mechanism, which spontaneous reporting data cannot establish. Tumor-type-specific ICI-naive reference cohorts were constructed for ACC and thyroid carcinoma. ICI-naive cohorts were not constructed for neuroendocrine tumor or pheochromocytoma/paraganglioma because their endocrine confounders do not directly involve the hypothalamic-pituitary-adrenal axis of interest, and subgroup sizes were insufficient for reliable confounding estimation. The ACC subgroup analysis was pre-specified as exploratory in view of its small ICI-exposed cell size; power considerations reported alongside the ACC results therefore serve to characterize the precision of the estimate rather than to support inferential claims. Demographic comparison used standardized mean differences (SMD; balance threshold <0.10). Multivariable logistic regression adjusted for age and sex estimated the association between ICI exposure and adrenal insufficiency within each tumor-type subgroup. Because the thyroid carcinoma reference cohort had a low background event rate, and because such sparse configurations can destabilize maximum-likelihood estimates, the thyroid carcinoma model was repeated using Firth penalized logistic regression. To address the concern that reporter type may influence both demographic completeness and event reporting, the model was refitted with a healthcare-professional reporter indicator (physician, health professional, or pharmacist versus other) as an additional covariate. Robustness to missing demographic data was assessed in two ways: through multiple imputation of missing age (20 imputations, predictive mean matching, estimates combined by Rubin rules), and through a tipping-point analysis that quantified how far the adrenal insufficiency rate among demographically incomplete records would have to depart from the observed rate in order to move the lower confidence bound of the thyroid carcinoma association below 1.0. As an alternative to the stratified reference design, a single combined model was fitted on the pooled ACC and thyroid carcinoma data with an ICI-by-tumor-type interaction term, both by standard and by Firth penalized logistic regression, to test whether the modifying effect of tumor type was recoverable within one model. We also extended the tumor-type-specific evaluation to the remaining endocrine irAE categories where event counts permitted stable estimation.

### 2.6 Time-to-onset analysis

Time-to-onset (TTO) was defined as days from ICI initiation to adverse event onset. Records with TTO = 0 (n = 148) or TTO <0 were excluded as non-informative, and records with TTO >1,095 days (3 years) were excluded because events first reported beyond this window are unlikely to reflect a plausible causal relationship to ICI initiation and are more susceptible to protopathic and recall bias. Kaplan–Meier cumulative incidence curves were compared using log-rank tests. Weibull parametric models were fitted via survreg, with the shape parameter k estimated alongside 95% CIs by the delta method. The Weibull family was chosen because it accommodates monotonically increasing, decreasing, or constant hazards through a single interpretable shape parameter, which directly addresses the clinical question of whether irAE risk concentrates early or accrues over time; an exponential model was not used because it constrains the hazard to be constant, and the log-normal and log-logistic alternatives, which impose non-monotonic hazards, were less consistent with the observed cumulative-incidence shapes and gave higher Akaike information criterion (AIC) values. Model fit was assessed by comparing AIC across these parametric families and by visual inspection of the fitted versus Kaplan–Meier curves. Shape parameter interpretation: k > 1.1 indicates increasing hazard; k < 0.9 decreasing hazard; k = 0.9 to 1.1 approximately constant hazard. Because TTO in FAERS reflects time to reporting rather than verified biological onset, the Weibull shape parameters are interpreted as descriptive of reported-event kinetics rather than as validated hazard functions.

### 2.7 Sensitivity analyses

Two sensitivity analysis sets were conducted. First, tyrosine kinase inhibitor (TKI) co-exposure exclusion and calendar-period stratification (2015 to 2019 vs. 2020–2024). Second, five pharmacologic proxy exclusion scenarios approximating surgical and medication history not captured in FAERS: exclusion of thyroid hormone replacement (post-thyroidectomy proxy), adrenal hormone replacement (pre-existing adrenal insufficiency proxy), systemic corticosteroids at immunosuppressive doses, and all three combined. These represent robustness checks rather than definitive confounding adjustments.

## 3 Results

### 3.1 Cohort characteristics

Following deduplication, 80,520 ICI-exposed reports were identified. The case cohort comprised 329 patients with endocrine cancers and the control cohort 80,191 patients (Table 1). Median age was 64 years (IQR, 54–72) in the case cohort and 65 years (IQR, 56–72) in controls (p = 0.037). Female sex was more prevalent in the case cohort (49.8% vs. 39.3%; p = 0.0002). Healthcare professionals contributed the majority of reports in both cohorts (case 70.2%, control 72.9%), with patient or consumer reports making up a smaller share (case 13.1%, control 11.3%); the remaining reports came from other or unspecified reporter types. Given the very large control cohort, these between-cohort differences reached statistical significance despite being small in absolute terms (for example, a one-year difference in median age), and we therefore interpret them as statistically detectable rather than clinically meaningful; they were nonetheless carried forward as adjustment covariates in the confounding analysis.

**TABLE 1:** Baseline demographic characteristics.

Characteristic	Case cohort (n = 329)	Control cohort (n = 80,191)	p-value
Median age, years (IQR)	64 (54–72)	65 (56–72)	0.037
Female sex, n (%)	164 (49.8%)	31,515 (39.3%)	0.0002
Age missing, n (%)	43 (13.1%)	17,114 (21.3%)	—
Sex missing/unknown, n (%)	28 (8.5%)	9,650 (12.0%)	—
Reporter — healthcare professional	231 (70.2%)	58,441 (72.9%)	—
Reporter — patient/consumer	43 (13.1%)	9,033 (11.3%)	—

*IQR*, interquartile range; Reporter categories follow FAERS, occupation codes, with healthcare professional defined as physician, other health professional, or pharmacist, and patient/consumer as consumer; remaining reports were of other or unspecified reporter type. Missing data reflect FAERS, reporting characteristics, and complete-case analysis was applied under a Missing at Random assumption (Section 2.2).

### 3.2 Primary disproportionality analysis

**FIGURE 1:**
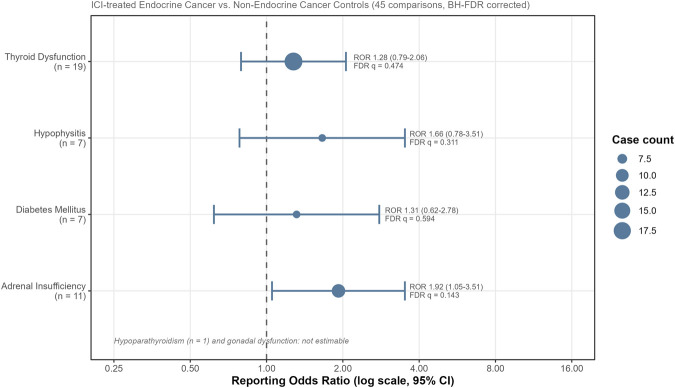
Primary disproportionality analysis. Forest plot of reporting odds ratios (ROR) with 95% confidence intervals for the pre-specified endocrine irAE categories. Dashed vertical line marks the null reference (ROR = 1.0). FDR-corrected q-values are shown adjacent to each estimate. No category reached the FDR significance threshold (q < 0.05) in the pooled analysis. Hypoparathyroidism and gonadal dysfunction were not estimable owing to small counts.

This pooled analysis compared the 329 ICI-treated endocrine cancer cases against the 80,191 ICI-treated non-endocrine cancer controls. In this comparison, adrenal insufficiency yielded the highest point estimate among the six categories (ROR = 1.922; 95% CI, 1.051–3.513), but this did not survive Benjamini–Hochberg correction across the 45-comparison family (Fisher p = 0.054; FDR q = 0.143). No endocrine irAE category reached the FDR significance threshold in the pooled analysis. Thyroid dysfunction, overall-cohort hypophysitis, and diabetes mellitus showed no disproportionate reporting, and hypoparathyroidism and gonadal dysfunction were not estimable because of small counts. Results are presented in Table 2; Figure 1. The absence of a significant pooled signal is itself informative: as shown in Sections 3.5, 3.6, this pooled non-significance masked two opposing tumor-type-specific patterns that only the confounding-aware reanalysis brought out.

**TABLE 2:** Primary disproportionality analysis.

irAE category	n	ROR (95% CI)	IC_025_	FDR q	Signal
Adrenal insufficiency	11	1.922 (1.051–3.513)	>0	0.143	Negative
Hypoparathyroidism	1	NE	NE	NE	Insufficient n
Diabetes mellitus	7	1.314 (0.620–2.784)	<0	0.594	Negative
Hypophysitis	7	1.658 (0.782–3.514)	<0	0.311	Negative^†^
Thyroid dysfunction	19	1.277 (0.793–2.057)	<0	0.474	Negative
Gonadal dysfunction	3	NE	NE	NE	Insufficient n

n denotes the number of case-cohort reports for each category. No category reached the FDR, significance threshold (q < 0.05) in the pooled analysis.

^†^ Drug class-stratified analysis identifies a significant anti-CTLA-4, hypophysitis signal (Section 3.4). NE, not estimable; IC_025_, lower bound of the 95% Bayesian credibility interval. Adrenal insufficiency was defined by the standardized term set described in Section 2.4.

### 3.3 Time-to-onset analysis

**FIGURE 2:**
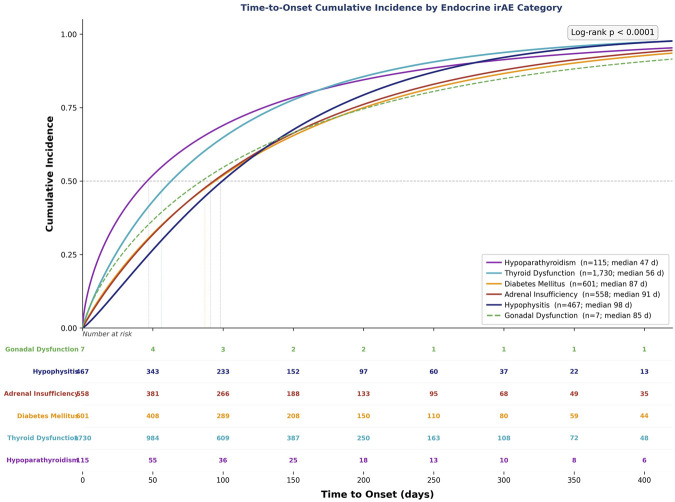
Kaplan–Meier cumulative incidence curves and number-at-risk tables by endocrine irAE category. Log-rank p < 2 × 10^−16^. Dashed horizontal line marks 50% cumulative incidence; dotted vertical lines indicate category-specific median time-to-onset. Gonadal dysfunction (n = 7) is shown for completeness but excluded from parametric interpretation.

Time-to-onset distributions differed significantly across irAE categories (log-rank p < 2 × 10^−16^; Figure 2). Median TTO was shortest for hypoparathyroidism (47 days; n = 115), followed by thyroid dysfunction (56 days; n = 1,730), diabetes mellitus (87 days; n = 601), adrenal insufficiency (91 days; n = 558), and hypophysitis (98 days; n = 467). Weibull modeling identified hypophysitis as the sole irAE category with an increasing hazard trajectory (k = 1.175; 95% CI, 1.096 to 1.260; AIC = 5,453.2). Hypoparathyroidism (k = 0.672) and thyroid dysfunction (k = 0.889) exhibited decreasing hazard consistent with early-concentrated risk. Adrenal insufficiency demonstrated an approximately constant hazard (k = 0.952; 95% CI, 0.892–1.016), with the CI spanning 1.0 precluding definitive classification.

### 3.4 Drug class-stratified analysis

**FIGURE 3:**
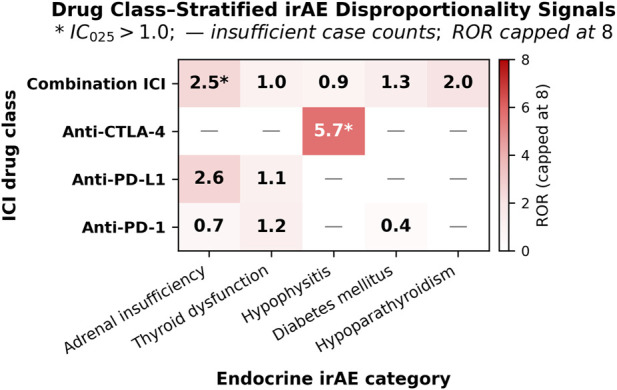
Drug class-stratified irAE signals. Heatmap of ROR values across four ICI exposure categories (combination ICI, anti-CTLA-4, anti-PD-L1, anti-PD-1) and five endocrine irAE categories. Color scale 0–8 (capped). Asterisks denote IC_025_ > 1.0. Anti-CTLA-4 hypophysitis (ROR = 5.7*) and combination ICI adrenal insufficiency (ROR = 2.5*) were the only statistically significant signals. Em dashes indicate cells with insufficient case counts for stable estimation.

Anti-CTLA-4 therapy demonstrated the strongest drug class-specific signal across all comparisons, with hypophysitis achieving the highest ROR in the entire analysis (ROR = 5.70; FDR-corrected p < 0.05). This was the sole drug class–irAE combination surviving Benjamini–Hochberg correction (Figure 3). Combination anti-PD-1/PD-L1 plus anti-CTLA-4 was the only regimen yielding significant adrenal insufficiency disproportionality (ROR = 2.51; IC_025_ > 1.0), whereas anti-PD-1 monotherapy did not reach signal threshold (ROR = 0.76). The anti-CTLA-4 hypophysitis signal provides internal methodological validity by recovering a mechanistically established association (Iwama et al., 2014).

### 3.5 Tumor-type subgroup analyses

**FIGURE 4:**
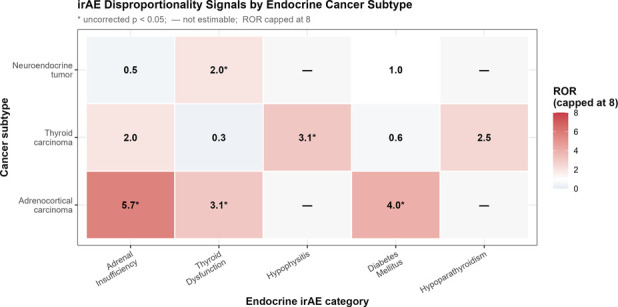
irAE signals by endocrine cancer subtype. Heatmap of uncorrected ROR values across three cancer subtypes (adrenocortical carcinoma, thyroid carcinoma, neuroendocrine tumor) and five endocrine irAE categories. Asterisks denote uncorrected p < 0.05. The strongest uncorrected subgroup signal was adrenal insufficiency in ACC (uncorrected p = 0.007), which the confounding-adjusted analysis (Section 3.6) showed to be most consistent with disease-inherent pathophysiology rather than ICI-attributable toxicity. None of the uncorrected subgroup signals survived FDR correction.

Tumor-type stratified signals are presented in Figure 4. Among the uncorrected subgroup comparisons, adrenal insufficiency in ACC was the strongest single signal (Fisher p = 0.007; FDR q = 0.072), and thyroid carcinoma showed uncorrected positive signals for both adrenal insufficiency and hypophysitis. In the neuroendocrine tumor subgroup, thyroid dysfunction yielded an uncorrected signal. None of these uncorrected subgroup signals survived FDR correction across the full comparison family. Because these are unadjusted reporting comparisons that do not account for the differing background event rates of each tumor type, they are presented descriptively; the formal confounding-adjusted analysis for ACC and thyroid carcinoma, which is the basis for our interpretation, is presented in Section 3.6.

### 3.6 Tumor-type-specific confounding analysis

In ICI-exposed versus ICI-naive ACC patients, demographic balance was adequate (age mean 46.0 vs. 47.1 years; SMD = 0.056; sex SMD = 0.144). In the pooled subgroup analysis, adrenal insufficiency in ACC had been the strongest uncorrected signal (Fisher p = 0.007; FDR q = 0.072), which in a conventional reading would suggest an ICI-related effect. After adjustment for age and sex, however, ICI exposure was not associated with adrenal insufficiency in ACC (adjusted OR = 0.58; 95% CI, 0.09–1.95), and a Firth penalized model gave a concordant estimate (OR = 0.71; 95% CI, 0.15–2.17). We emphasize that this subgroup rests on only four ICI-exposed events, and that with an estimated 12.6% power the confidence interval spans both substantial protection and a near-doubling of odds. The analysis is therefore insufficient to confirm or exclude an association in either direction, and we do not interpret the non-significant estimate as evidence of no effect. What can be said is that ICI-exposed ACC patients reported adrenal insufficiency at a prevalence similar to ICI-naive patients (9.3% vs. 8.6%), a pattern that, together with the autonomous glucocorticoid hypersecretion and mitotane-induced adrenolysis intrinsic to ACC, makes a disease-inherent explanation more parsimonious than a drug-attributable one.

In the thyroid carcinoma subgroup, sex was well balanced (SMD = 0.064). Here the adjusted analysis pointed in the opposite direction to ACC. ICI exposure was associated with markedly higher adrenal insufficiency reporting odds (adjusted OR = 13.33; 95% CI, 4.81–31.85), with a null age coefficient (OR per year = 1.000; 95% CI, 0.975–1.030) indicating no age-related confounding. Because the ICI-naive background rate was low (0.28%) and the exposed subgroup small (n = 172, six events), we tested this association against several threats to its stability. The estimate was essentially unchanged after adding a healthcare-professional reporter indicator (adjusted OR = 13.23), under Firth penalized regression for sparse data (OR = 14.06; 95% CI, 5.28–32.80), and under multiple imputation of missing age across the full subgroup (OR = 12.89; 95% CI, 5.26–31.60). A tipping-point analysis showed that the demographically incomplete records excluded from the complete-case model would need to carry an adrenal insufficiency rate of roughly 1.8% (about six times the observed 0.28%) before the lower confidence bound crossed 1.0, a degree of outcome-correlated missingness that spontaneous reporting data do not support. The wide confidence interval reflects the subgroup size, and the point estimate should be read as evidence of a strong positive disproportionality signal rather than as a precise risk magnitude. We address the residual role of differential surveillance in Section 4. These results are summarized in Table 3.

**TABLE 3:** Tumor-type-specific confounding analysis.

Tumor	irAE	ICI-exposed	ICI-naive	Adj. OR (95% CI)	p	Power
ACC	Adrenal insufficiency	9.3% (4/43)	8.6% (114/1317)	0.58 (0.09–1.95)	NS	12.6%
Thyroid Ca	Adrenal insufficiency	3.5% (6/172)	0.28% (28/10069)	13.33 (4.81–31.85)	<0.001	—

Adjusted OR, from multivariable logistic regression (age + sex). ACC, adrenocortical carcinoma; Ca., carcinoma; NS, not significant; SMD, balance threshold <0.10. Post-hoc power values are reported descriptively to characterize estimate precision (Sections 2.5, 3.6); the ACC, analysis is pre-specified as exploratory.

Extending the tumor-type-specific evaluation to the remaining endocrine irAE categories, hypophysitis in thyroid carcinoma was reported only among ICI-exposed patients (7 of 172) and in none of the 10,069 ICI-naive patients. This complete separation precludes a stable odds ratio, so we report it as a qualitative observation rather than a point estimate; its direction is consistent with an ICI-related effect and supports the possibility that part of the adrenal insufficiency signal in this subgroup is central in origin. Thyroid dysfunction in thyroid carcinoma showed no association with ICI exposure (Firth OR = 0.80; 95% CI, 0.22–2.01), as expected if thyroid dysfunction in these patients is largely disease- and treatment-inherent. The remaining categories (diabetes, hypoparathyroidism, gonadal dysfunction) had too few events for stable subgroup estimation. As an alternative to the stratified design, a single pooled model with an ICI-by-tumor-type interaction reproduced the same structure: the main ICI term (in ACC) was near null, while the ICI-by-thyroid interaction was strongly positive and significant (Firth interaction OR = 19.6; 95% CI, 4.4–116.7), confirming within one model that the effect of ICI exposure on adrenal insufficiency reporting differs by tumor type. Notably, a covariate-only model that included tumor type without an interaction term returned an ICI coefficient close to the pooled disproportionality estimate (OR = 1.92), illustrating how ignoring effect modification reproduces the uninformative pooled average.

### 3.7 Sensitivity analyses

The pooled adrenal insufficiency point estimate was stable across the sensitivity scenarios (Table 4). Sequential exclusion of pharmacologic proxies for unmeasured surgical and medication history (thyroid hormone replacement, adrenal hormone replacement, and systemic corticosteroids, individually and combined) did not attenuate the estimate, which remained in the same range as the main analysis (ROR 1.83–2.12) rather than moving toward the null; this argues against the pooled signal being driven by these disease- or treatment-related confounders. Exclusion of TKI-exposed reports likewise did not weaken the estimate (ROR = 2.931; 95% CI, 1.501–5.721). Calendar-period stratification showed a higher early-period estimate that attenuated in the later period, consistent with denominator expansion as ICI prescribing broadened rather than with a true change in risk.

**TABLE 4:** Adrenal insufficiency ROR across sensitivity scenarios.

Scenario	Case n	Control n	ROR	95% CI
Main analysis	329	80,191	1.922	1.051–3.513
Excl. TKI-exposed	235	80,191	2.931	1.501–5.721
1. Excl. Thyroid hormone replacement	299	80,191	2.122	1.159–3.883
2. Excl. Adrenal hormone replacement	314	80,191	1.827	0.971–3.438
3. Excl. Systemic corticosteroids	300	80,191	1.916	1.018–3.606
4. Excl. All three proxies	279	80,191	2.065	1.096–3.891

All scenarios yielded IC_025_ > 1.0, indicating statistically significant positive signals.

### 3.8 Temporal and outcome analyses

**FIGURE 5:**
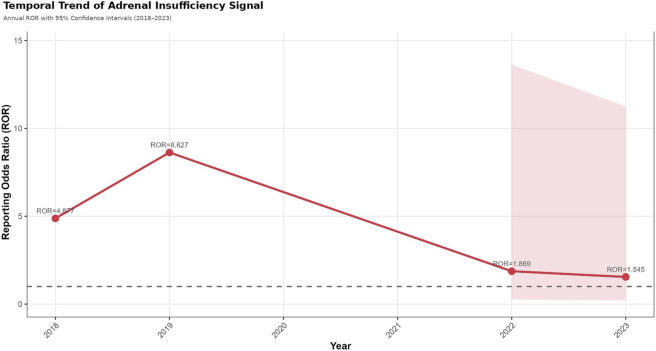
Temporal trend of the adrenal insufficiency pharmacovigilance signal across the study window. Annual reporting odds ratio with 95% confidence interval where estimable (shaded band). Dashed horizontal line marks the null reference (ROR = 1.0). The signal rose to a peak around 2019 and progressively attenuated thereafter, a pattern most consistent with denominator expansion as ICI prescribing volumes grew rather than with a true change in underlying risk.

**FIGURE 6:**
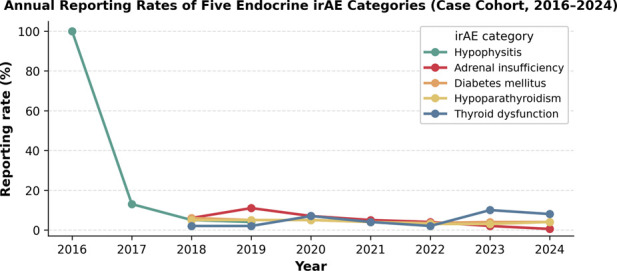
Annual reporting rates of the five quantitatively analyzed endocrine irAE categories in the case cohort across the 2016 to 2024 study window. The 2016 hypophysitis value of 100% reflects a single early case in a minimal denominator and is shown for completeness rather than as a stable estimate. From 2018 onward, all categories settled within a 0%–10% range, with thyroid dysfunction exhibiting the only sustained upward drift after 2022 (consistent with the Spearman trend statistic reported in the text).

The annual adrenal insufficiency reporting odds ratio followed a non-monotonic trajectory, rising to a peak around 2019 and attenuating thereafter (Figure 5), a pattern consistent with denominator expansion as ICI prescribing volumes grew rather than with a true change in underlying risk. Thyroid irAE signals showed a significant positive temporal trend (Spearman rho = 0.83; p = 0.006), while hypophysitis showed a significant negative trend (rho = −0.91; p = 0.001). The full annual reporting-rate trajectory of the five quantitatively analyzed endocrine irAE categories across the 2016 to 2024 window is shown in Figure 6. The apparent dominance of hypophysitis in 2016 reflects a single early case in a very small denominator and normalized rapidly as prescribing volumes expanded, settling within a 0%–10% band from 2018 onward. Thyroid dysfunction showed the only sustained upward drift, rising modestly after 2022, which is coherent with the Spearman statistic and with broader integration of thyroid function monitoring into routine ICI follow-up. Adrenal insufficiency, diabetes mellitus, and hypoparathyroidism stayed within a narrow 0%–10% band throughout. Among adrenal insufficiency cases, 90% required hospitalization and 30% were classified as life-threatening, underscoring the clinical seriousness of the events behind these reports.

## 4 Discussion

The central contribution of this pharmacovigilance analysis is methodological. In the pooled case-versus-control comparison, adrenal insufficiency did not reach the disproportionality signal threshold after FDR correction (ROR = 1.922; FDR q = 0.143). A conventional reading would stop there and conclude that the data carry no actionable endocrine signal. When tumor-type-specific ICI-naive reference populations are introduced, however, this single uninformative pooled estimate resolves into two opposing phenomena: a subgroup (ACC) in which the strongest uncorrected signal does not survive demographic adjustment and is most consistent with disease-inherent pathophysiology, and a subgroup (thyroid carcinoma) in which a strong and robust positive association is present that the pooled analysis had averaged away. Pooling therefore misleads in both directions at once, generating an apparent signal where adjustment removes it and masking a genuine one where adjustment reveals it. The practical consequence is that regulators, clinicians, and investigators relying on pooled FAERS signals in heterogeneous endocrine cancer populations may both act on artefacts and overlook real, potentially actionable associations.

In thyroid carcinoma the confounding-aware analysis identifies a strong positive disproportionality signal that we interpret as hypothesis-generating rather than as a confirmed causal effect. The multivariable-adjusted association between ICI exposure and adrenal insufficiency reporting (adjusted OR = 13.33; 95% CI, 4.81–31.85) was not explained by demographic confounding, and it held under three independent robustness checks: addition of reporter type (OR = 13.23), Firth penalized regression for sparse data (OR = 14.06), and multiple imputation for missing demographics (OR = 12.89). A tipping-point analysis indicated that only an implausibly extreme departure of the unobserved records from the observed event rate could nullify it. The most important caveat is differential ascertainment: ICI-treated patients undergo structured endocrine surveillance whereas the ICI-naive reference patients do not, so subclinical adrenal insufficiency is more likely to be detected and reported in the exposed group, and this unmeasured difference may account for part of the association. For this reason the odds ratio should be read as evidence of a substantial signal rather than as a precise effect size. Several non-mutually-exclusive mechanisms may also contribute. ICI-induced hypophysitis producing isolated ACTH deficiency and central hypoadrenalism is biologically plausible given the co-occurrence of both adrenal insufficiency and hypophysitis signals in the thyroid carcinoma subgroup, and is supported by case-level documentation of nivolumab-associated concurrent thyroiditis and isolated ACTH deficiency in a patient carrying autoimmune polyendocrine syndrome susceptibility HLA haplotypes (Ohara et al., 2019). ICI-amplified polyglandular autoimmune syndrome type 2 represents a second pathway: susceptibility loci including HLA-DR3/DQ2, HLA-DR4/DQ8, and the CTLA-4 gene on chromosome 2 (Kahaly, 2009; Van den Driessche et al., 2009) are the same targets as ipilimumab, and Hashimoto thyroiditis coexists with papillary thyroid carcinoma in approximately 19% of surgical cases (Xu et al., 2021), sharing HLA susceptibility alleles with adrenal autoimmunity. Third, ICI-induced destructive thyroiditis, via its thyrotoxic phase, may impose increased cortisol demand on patients with attenuated adrenocortical reserve, precipitating adrenal decompensation without primary adrenal destruction. Clinically, these findings support a low threshold for biochemical evaluation of both primary and central adrenal insufficiency in ICI-treated thyroid carcinoma patients, including early morning cortisol, ACTH, and dynamic testing with ACTH stimulation. Prospective validation incorporating HLA genotyping and pituitary MRI surveillance is warranted.

In ACC the analysis points in the opposite direction, although here the data are limited and we are careful not to overstate them. Adrenal insufficiency in ACC had been the strongest uncorrected subgroup signal, which a conventional reading might attribute to ICI exposure, yet it did not survive adjustment for age and sex (adjusted OR = 0.58; 95% CI, 0.09 to 1.95; Firth OR = 0.71). We do not read this as evidence that ICI exposure has no effect on adrenal insufficiency in ACC. With only four exposed events and approximately 12.6% power, the analysis is simply insufficient to confirm or exclude an association, and the confidence interval is compatible with both meaningful protection and a near-doubling of odds. What the data do show is that ICI-exposed and ICI-naive ACC patients reported adrenal insufficiency at similar prevalence (9.3% versus 8.6%). Combined with the autonomous glucocorticoid hypersecretion and mitotane-induced adrenolysis that characterize ACC (Fassnacht et al., 2018; Grouthier et al., 2020), and with the consistently modest antitumor activity of ICIs in advanced ACC attributable to an immunoevasive, low-mutational-burden phenotype (Raj et al., 2020; Habra et al., 2019; Klein et al., 2021; Yarchoan et al., 2017), a disease-inherent explanation for adrenal insufficiency in this setting is more parsimonious than a drug-attributable one. The clinical implication is cautionary rather than conclusive: adrenal insufficiency in an ICI-treated ACC patient should not be automatically attributed to ICI toxicity, and disease-inherent and mitotane-related mechanisms should be considered in the differential before ICI discontinuation, which could otherwise deprive the patient of active therapy on the basis of a misattributed event.

The framework presented here extends established concerns regarding comparator selection in spontaneous reporting analysis (van Puijenbroek et al., 2002; Bate et al., 1998). Whereas conventional disproportionality treats the database as a self-referential reference set, the channeling of specific drugs into specific disease populations can produce structural confounding analogous to confounding by indication in observational pharmacoepidemiology. Our tumor-type-specific reference design represents an operational instantiation of the active comparator paradigm adapted for spontaneous reporting data, and complements rather than replaces conventional pooled disproportionality.

The anti-CTLA-4 hypophysitis signal (ROR = 5.70; FDR-corrected p < 0.05) is the sole drug class–irAE association surviving FDR correction across 45 comparisons, and serves as internal methodological validation of our pipeline (Figure 3). This finding is well-grounded mechanistically: pituitary cells constitutively express CTLA-4, rendering them susceptible to complement-mediated type II hypersensitivity when anti-CTLA-4 antibodies bind to ectopically expressed antigen, a mechanism demonstrated by C3d and C4d complement deposition at sites of pituitary inflammation (Iwama et al., 2014). The restriction of significant adrenal insufficiency disproportionality to combination ICI therapy, with anti-PD-1 monotherapy carrying no signal, is consistent with the additive immunotoxicity of concurrent checkpoint blockade (Barroso-Sousa et al., 2018). We note that combination ICI recipients likely have better performance status and more intensive clinical monitoring than monotherapy recipients, and this ascertainment differential cannot be adjusted for within a spontaneous reporting framework; it represents a plausible secondary contributor to the observed signal magnitude.

Weibull parametric modeling identifies hypophysitis as the sole irAE category with an increasing hazard trajectory, suggesting that pituitary irAE risk does not plateau during ICI treatment. This pattern is mechanistically coherent with cumulative complement-mediated corticotroph destruction under sustained anti-CTLA-4 exposure (Iwama et al., 2014), and has a practical monitoring corollary: routine early-treatment endocrine surveillance may be insufficient for hypophysitis detection in anti-CTLA-4 recipients, and late-treatment pituitary vigilance should be maintained. The markedly decreasing hazard functions for hypoparathyroidism (k = 0.672) and thyroid dysfunction (k = 0.889) indicate risk concentrated in the early treatment period, supporting front-loaded monitoring for these events. The approximately constant adrenal hazard trajectory, with the CI spanning 1.0, offers no basis for preferential surveillance timing and argues for sustained vigilance throughout ICI exposure. These kinetic observations, if prospectively confirmed, could inform irAE-specific monitoring schedules.

The annual reporting trajectories shown in Figure 6 deserve a brief interpretive note. The early hypophysitis spike of 2016 represents a single notoriety-driven case in an essentially absent denominator and should not be over-read; the rapid normalization from 2018 onward reflects denominator expansion rather than true biological attenuation, mirroring the temporal pattern of the adrenal insufficiency ROR in Figure 5. The mild upward drift of thyroid dysfunction reporting after 2022 is the only category-specific temporal signal that survives this denominator-expansion lens and is plausibly attributable to broader integration of thyroid function panels into standard ICI follow-up rather than to a genuine increase in incidence. Convergence of all five categories into a narrow 0%–10% reporting band by 2024 is consistent with the maturation of FAERS endocrine-irAE reporting practice and provides reassurance that none of our key findings are driven by an outlier reporting year.

The broader methodological implication of these findings is that confounding-aware pharmacovigilance, defined as disproportionality analysis paired with tumor- or disease-specific reference populations and explicit demographic adjustment, merits broader application whenever the adverse event of interest overlaps biologically with the disease under study. This applies beyond endocrine cancers: analogous concerns arise for hepatic irAE signals in hepatocellular carcinoma populations exposed to atezolizumab/bevacizumab, for cardiac irAE signals in cardio-oncology populations with pre-existing cardiomyopathy, and for renal irAE signals in urothelial carcinoma patients with baseline renal dysfunction. The analytic template applied here is generalizable to any disease–drug–adverse-event triad with biological overlap.

Our findings sit alongside an emerging FAERS-based literature on ICI endocrine toxicity that has thus far relied on pooled reference cohorts (Raschi et al., 2019; Zhai et al., 2019; Darapu et al., 2025; Mytareli et al., 2023), and they refine the published case-level descriptions of central adrenal insufficiency under PD-1 blockade (Hashinokuchi et al., 2023) by providing population-level signal decomposition. The interpretation of the thyroid carcinoma signal is further supported by the documented association of Hashimoto thyroiditis with papillary thyroid carcinoma (Xu et al., 2021; Villagelin et al., 2011; Feldt-Rasmussen, 2020; Noureldine and Tufano, 2015) and by the established pivotal role of ipilimumab in defining the modern checkpoint inhibitor toxicity profile (Hodi et al., 2010).

Several limitations warrant transparent acknowledgment. First, FAERS data are subject to underreporting, notoriety bias, and absence of denominator exposure data, precluding absolute incidence estimation (Sakaeda et al., 2013; Raschi et al., 2019). The ROR quantifies reporting disproportionality relative to database background and should not be interpreted as a clinical incidence estimator, a constraint that applies equally to our confounding-aware analyses. Second, the case cohort of 329 patients limits statistical power for rarer tumor-type subgroups; neuroendocrine tumor and pheochromocytoma/paraganglioma analyses are presented as exploratory only. Third, multivariable models adjusted for age and sex; residual confounding from disease stage, mitotane exposure, radioiodine history, and prior systemic therapy cannot be excluded, although pharmacologic proxy-exclusion sensitivity analyses did not attenuate the pooled adrenal insufficiency estimate, arguing against these factors as the primary driver of the pooled signal. Fourth, the thyroid carcinoma odds ratio should be interpreted as evidence of a substantial signal rather than a precise effect estimate, given the subgroup size and confidence interval width. Fifth, the MAR assumption underlying complete-case analysis is formally untestable in FAERS, and its violation would bias estimates in directions that depend on the missingness mechanism; we examined this through multiple imputation and a tipping-point analysis, both of which left the thyroid carcinoma association essentially unchanged, but a Missing Not at Random mechanism cannot be fully excluded. A further limitation specific to the thyroid carcinoma signal is differential ascertainment between ICI-treated and ICI-naive patients, discussed above, which adjustment for reporter type addresses only in part and which may contribute to the magnitude of the association. Sixth, *post hoc* power values are reported descriptively to characterize estimate precision and do not provide inferential warrant independent of the confidence interval and p-value. Seventh, pharmacological proxy exclusion is a workaround for unmeasured surgical and prior-therapy history; it does not substitute for direct confounder adjustment. Eighth, notoriety bias may inflate early-period adrenal insufficiency reporting and complicates causal inference. Prospective registry-based studies incorporating systematic baseline endocrine phenotyping, HLA genotyping, and standardized longitudinal hormone profiling are required to validate the thyroid carcinoma signal and to resolve the outstanding mechanistic questions.

## 5 Conclusion

We show that conventional disproportionality analysis applied to endocrine cancer patients as a single homogeneous group can misclassify drug-attributable and disease-inherent endocrine adverse events. The pooled adrenal insufficiency signal did not reach significance after FDR correction, yet a confounding-aware framework using tumor-type-specific ICI-naive reference populations revealed two opposing patterns beneath it. In adrenocortical carcinoma the data, though limited in power, are most consistent with disease-inherent pathophysiology and do not support a drug-attributable effect. In thyroid carcinoma ICI exposure showed a strong positive association with adrenal insufficiency reporting that was robust to reporter type, to sparse-data penalization, and to imputation of missing data; we interpret this as a hypothesis-generating signal that warrants prospective validation, while recognizing that differential endocrine surveillance between exposed and unexposed patients may contribute to its magnitude. Anti-CTLA-4 hypophysitis was the single drug class-irAE association surviving FDR correction and supports the internal validity of the pipeline, and the Weibull-derived increasing hypophysitis hazard is consistent with sustained late-treatment pituitary risk in anti-CTLA-4 recipients. Clinically, adrenal insufficiency in an ICI-treated thyroid carcinoma patient should prompt evaluation for both primary and central causes, whereas adrenal insufficiency in an ACC patient should not be reflexively attributed to ICI toxicity without considering disease-inherent and mitotane-related mechanisms. Methodologically, confounding-aware pharmacovigilance merits broader application whenever the adverse event of interest overlaps biologically with the disease under study.

## Data Availability

The original contributions presented in the study are included in the article/Supplementary Material, further inquiries can be directed to the corresponding author.
