# Supplementary figures and images for: Confounding-aware disproportionality analysis reveals disease-inherent versus drug-attributable endocrine safety signals of immune checkpoint inhibitors
Source: Front Pharmacol. 2026 Jun 23;17:1868030. doi: 10.3389/fphar.2026.1868030 (PMC13337867; doi:10.3389/fphar.2026.1868030)

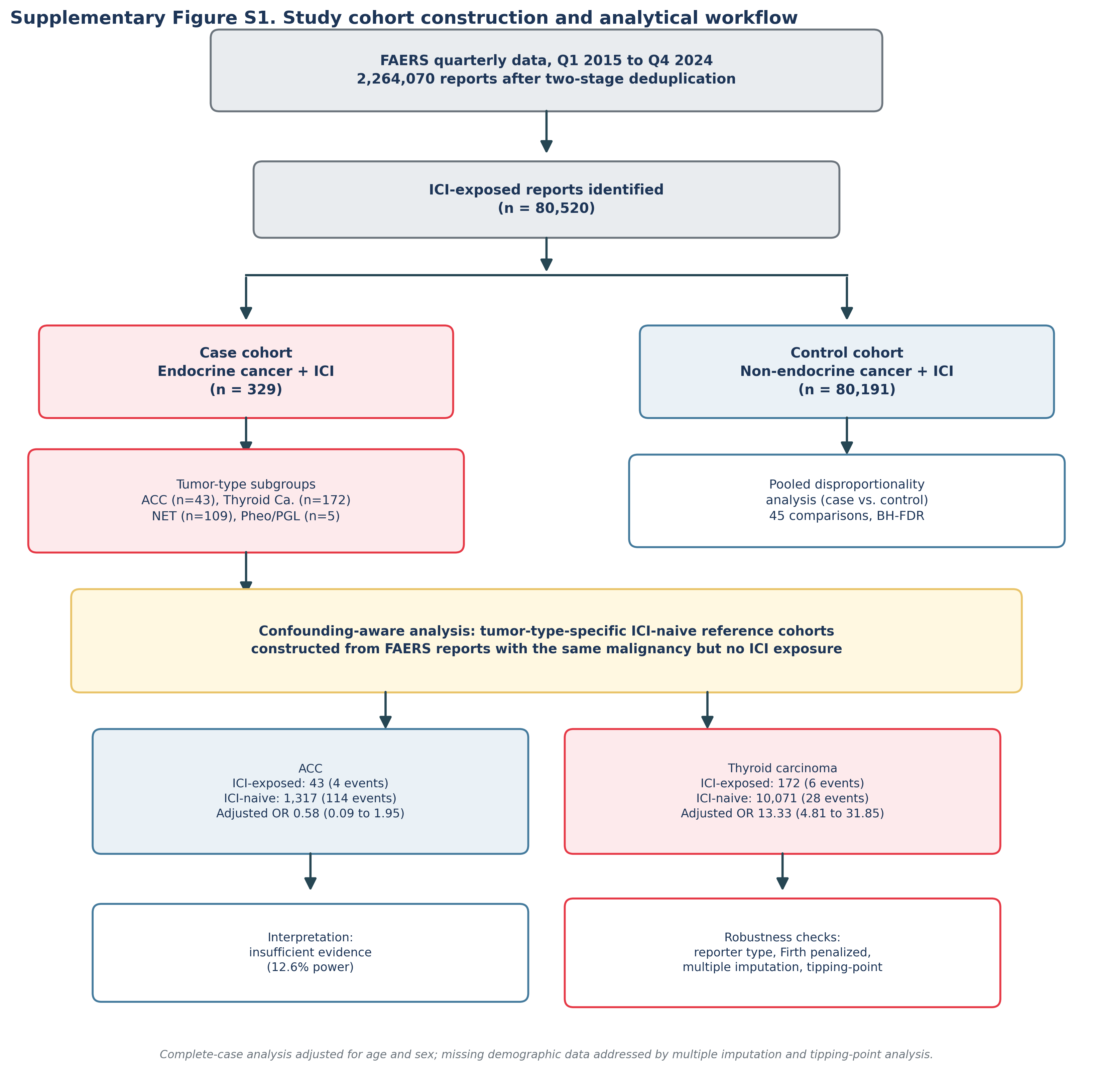

Supplement: Supplementary file 2 [file Image1.tif]
